# Generating Attributed Networks with Communities

**DOI:** 10.1371/journal.pone.0122777

**Published:** 2015-04-20

**Authors:** Christine Largeron, Pierre-Nicolas Mougel, Reihaneh Rabbany, Osmar R. Zaïane

**Affiliations:** 1 Hubert Curien Laboratory, University of Lyon, Saint-Étienne, France; 2 Department of Computer Science, University of Alberta, Edmonton, Canada; Universidad Rey Juan Carlos, SPAIN

## Abstract

In many modern applications data is represented in the form of nodes and their relationships, forming an information network. When nodes are described with a set of attributes we have an attributed network. Nodes and their relationships tend to naturally form into communities or clusters, and discovering these communities is paramount to many applications. Evaluating algorithms or comparing algorithms for automatic discovery of communities requires networks with known structures. Synthetic generators of networks have been proposed for this task but most solely focus on connectivity and their properties and overlook attribute values and the network properties vis-à-vis these attributes. In this paper, we propose a new generator for attributed networks with community structure that dependably follows the properties of real world networks.

## Introduction

A variety of present-day applications requires interrelated data with relationships between data points. The structures in these interrelated data are typically modeled by a graph of interconnected nodes, known as complex networks or sometimes social networks or information networks. Examples of such networks are citation or collaboration networks of scholars, or trust and social networks of humans. Although drawn from a wide range of domains, most real world networks exhibit common statistical properties, such as power law degree distribution, and small world characteristic, which enable implementing a generic set of techniques for analyzing these networks [1].

One fundamental property of many real networks is that they tend to organize according to an underlying modular structure [2]. These structures are known as communities and are commonly defined based on the dense connectivity between community members. Many community mining methods have been proposed and are typically based solely on relationships between nodes in the graph. Most of these methods validate their algorithms on standard benchmarks for which the true communities are known but there are few and typically small real world benchmarks with known communities available for this external evaluation of community detection algorithms. For this reason, synthetic benchmark generators have also been designed but the current generators overlook some characteristics of the real networks [3]. For example, the commonly used benchmarks in community mining evaluations, are the synthetic LFR benchmarks proposed in [4]. These benchmarks have been used as standard benchmarks in external evaluation and comparison of community mining results [5–8]. However, as noticed by [3, 9], the networks generated in these benchmarks fail to follow some very basic characteristics of real world networks, such as the densification power laws and heavy-tailed distribution. Moreover, the generators do not consider attributes of nodes. In real applications, the data points are more than merely vertices, they are accompanied with their attribute values, resulting in what is known as attributed networks [10] and, it has been shown that the communities depend both on relationships and attributes. This is the homophily effect [11]. In recent years, new approaches considering the attributes and the relationships to discover communities have been proposed. Nonetheless, their evaluation is a challenging issue since publicly available real attributed networks with known communities are rare and difficult to obtain. In addition, generators for synthetic attributed networks with communities are quasi non-existent.

Therefore we believe that there is a need for developing a more realistic benchmark generator to generate authentic networks with built-in community structures and attributed nodes, faithfully following the known properties of real-world attributed networks and closely ensuring intrinsic characteristics such as preferential attachment requiring nodes with high connectivity to have a higher chance to attract links with other nodes, and homophily, the tendency of nodes to associate with higher probability with nodes having similar attribute values. We propose herein a generator for attributed networks with community structure, show the parameters of the model and highlight some of the experiments to demonstrate reliability and flexibility of our model.

Section is dedicated to the state of the art, while the model is presented in Section. The experiments are described in Section and Section concludes.

## Related works

There are many generative network models proposed for real world networks [1, 12–15]. Here, we survey commonly used generative network models, focusing on models that incorporate built-in community structure, and/or attributes for the vertices.

### Networks without attribute or communities

The classical Erdős-Rényi (ER) model [16] generates random graphs of a given size, where edges are formed independently and with uniform probability. These graphs comply with the small-world property observed in real world graphs. However, they have a binomial degree distribution and therefore are not scale-free. The generated ER graphs also have a relatively low transitivity [17]. The Watts and Strogatz (WS) model [18] is another famous generator for small world graphs. This model starts with a regular graph (ring lattice), then rewires links with a probability *β*. The WS model is therefore able to generate small world graphs with high transitivity. The degree distribution, however, does not follow a power law, and therefore this model is not scale-free.

The Barabási-Albert (BA) model [19] is one of the most basic models that generates random scale-free networks. Starting with an initial network, vertices are added at each step, while the newly added vertex forms connections with the existing vertices according to the preferential attachment. Although the generated BA graphs comply with macroscopic properties observed in real networks, the evolution of networks in this model is not realistic as discussed by [20]. More specifically in the BA model, the probability of forming long-range edges is as likely as local edges, whereas in real networks edges are formed locally.

In [21, 22] the authors empirically observe that real networks become denser over time, with the average degree increasing and the diameter decreasing in many cases. They propose the Forest Fire (FF) model which exhibits these evolution patterns. In this model every new vertex first connects randomly to an existing vertex called ambassador. Then, it recursively forms a random number of connections with the neighbors of every vertex it connects to.

Other notable synthetic generators are the mathematical tractable models, such as the Stochastic Kronecker Graph model [23], and its generalization, the Multifractal Network model [24, 25]. These models generate networks with realistic properties, i.e., heavy-tailed degree distributions and high clustering coefficient, that can be mathematically proved. The recursive generation process is based on a set of generating parameters, i.e., hierarchical categories assigned to vertices that determine their probability of forming an edge. These parameters can be further fitted to a given real network.

### Networks with Communities

The GN benchmark [26] is the first network generator proposed for evaluating community mining algorithms. This benchmark is a graph of 128 vertices, with an expected degree of 16, and is divided into four groups of equal sizes where the probabilities of the existence of a link between a pair of vertices of the same group and of different groups are *z*_*in*_ and 1 − *z*_*in*_ respectively where *z*_*in*_ ∈ [0, 1]. Since all the vertices have the same expected degree and equal communities size, this model is not accordant to real social network properties.

LFR benchmark [4] amends the GN benchmark by distributing the degrees according to a power law. Similar to the GN benchmark, each vertex shares a fraction 1 − *μ* of its links with the other vertices of its community and a fraction *μ* with the other vertices of the network. An extension of this benchmark for overlapping communities is presented in [27].

The process of generating the commonly used LFR benchmarks [4] could be summarized into two steps. In the first step, it generates a random network which has a power law degree distribution by first assigning a degree to each vertex taken from the power law distribution, and then linking vertices randomly. In the second step, it imposes a community structure on the network by first determining the number of communities and their sizes, and then randomly assign vertices to the communities.

This generation process has two main issues. First, the original network is generated with a very simple network model, i.e., the configuration model [28]. Therefore, although the networks generated by this model follow the power law degree distribution, they fail to follow some other typical characteristics of real world networks. In particular, [29] shows that the generated LFR networks exhibit low transitivity and close to zero degree correlation. To remedy this they proposed two variants of the LFR benchmarks [30] by replacing the configuration model in the LFR with a more realistic network models, i.e., the preferential attachment model of [19] and the evolutionary preferential attachment model of [31]. These modified benchmarks have an improved realism, however they are less flexible as they have less parameters compared to the original LFR generators.

The second issue with the LFR benchmark generators is enforcing communities later on the network which is in contrary to their definition as the natural structure underlying the networks. Orman and Labatut also mention this fact that the rewiring process in general changes the network structure chaotically, but leave it untackled [3]. Moussiades and Vakali further describe this issue as choosing a random clustering as the ground truth [9].

The block two-level Erdős-Rényi (BTER) model proposed in [32, 33], directly incorporates communities in the generative model, whereas their networks are scale-free collections of ER subgraphs as communities. The BTER starts with a preprocessing where vertices are distributed into communities and each vertex is assigned a degree and a clustering coefficient (the latter determines the portion of between to within community links), which are input to the model. Then in phase 1, local links are formed within each community according to a constant probability computed for that community, and in the second phase, between edges connect communities together. If the input degree distribution follows a power law, the resulted networks are shown to be scale-free. The model presented in this article is similar to the BTER model, however, when forming long range edges between communities, it considers similarity of vertices in terms of their attributes.

### Networks with Attributes

Evidences of homophily in most real networks suggest that connections are formed with a bias in favor of similar attributes of vertices (including their degrees). Some network generator models assume that links between vertices are formed solely based on their attributes [34, 35].

To take into account this behavior, another possibility is to consider attributes as vertices within an augmented network. Social-Attribute Network (SAN) proposed by [36] follows this approach. They define SAN as a heterogeneous network consisting of vertices representing either individues or attribute values. The attribute links connect the binary attributes to vertices that posses that attribute value. They first empirically observe characteristics of such a model (e.g. attribute degree distribution, social degree distribution) in a real network dataset (Google+). Then they propose a generative process to synthesize SAN networks with similar characteristics following attribute-augmented preferential attachment (the probability of an edge between a vertex *u* and a vertex *v* depends on the degree of vertex *v* as well as the number of attributes *u* and *v* have in common) and attribute-augmented triangle-closing (randomly connecting *u* with its 2-hop social neighbors, where the hop could be through attribute vertices).

Another approach has been proposed in [37] using random typing. Each vertex corresponds to a weighted random sequence of characters where two vertices are more likely to be connected if they share a sequence of characters. This model allows to generate graphs having a realistic structure, however, it is not possible to build two distinct vertices having the same set of attributes. Moreover, the attributes are required to be discrete.

### Networks with Communities and Attributes

Very few generators allow one to build a network having both a community structure and attributes associated with the vertices. In [38] the author proposes a simple generation model in which for each new vertex, its attributes and community membership are independently sampled from a multinomial and normal distribution, respectively. It then forms specified number of edges, where the probability of linking to an existing vertex depends on the multiplication of (1) the degree of that vertex, (2) the attribute similarity of that vertex to the new vertex and (3) the attribute similarity of the class of the first vertex to the class of the second vertex. While this approach allows to accurately model several real networks properties, the homophily property is somehow biased due to the multinomial distribution of the attributes. Indeed, vertices in a given community are more likely to connect to only a few other communities (those having a similar mean for the normal distribution), and will not being connected to most other communities.

While not being dedicated to the generation of networks, several articles [39, 40] have proposed graph models of networks with attributes. Given an existing graph, the objective is to discover parameters of the model fitting the real network structure and attribute distribution. These latent parameters are used to infer knowledge from the graph, e.g., the community structure. However, while not being a straightforward task, these models can also be used to generate a new graph similar to an existing one using the discovered model parameters. A study remains to be done to find which networks characteristics are captured by the models. In [39] the authors propose a generative Bayesian model to learn the latent parameters of attribute models and communities given the fact that graph and attributes of vertices are observed and independent given community structure. Similarly, [40] propose a generative Bayesian model for sampling clustered attributed networks, and infer clusters in such networks based on a variational approximation approach.

Given the interest of discovering communities in a social network where vertices are associated with attributes, we believe that a generator of networks following real networks properties and allowing to assess the quality of the obtained results using a ground truth is valuable.

## Model

### Hypothesis

We provide a new attributed network with community structure generator. Such networks can be represented by an attributed graph 𝓖 = (𝓥,𝓔,𝓐), where 𝓥 is a set of vertices, 𝓔 a set of edges and 𝓐 a set of real attributes associated with the vertices such that each vertex *v* ∈ 𝓥 is described by an attribute value vector *v*_*A*_[10].

The network has a community structure if the nodes are grouped into sets densely connected and relatively homogeneous regarding the attributes. We suppose that these communities are not overlapping and consequently, they define a partition 𝓟 of 𝓥 such that (1) ∀(*C*_1_, *C*_2_) ∈ 𝓟×𝓟, *C*_1_∩*C*_2_ = ∅; (2) ∀*C* ∈ 𝓟,*C* ≠ ∅; and (3) $\underset{C\in 𝓟}{\cup }C=𝓥$.

The proposed attributed network with community structure generator is based on the following hypothesis.

It respects the preferential attachment according to which new nodes prefer to join to the more connected nodes existing in the network. Thus, each node is connected to an existing node with a probability proportional to the number of links of the chosen node. Given this hypothesis, our model can be considered as an extension of the Barabasi-Albert model: it leads to scale-free networks characterized by a degree distribution with a heavy tail which can be approximated by a power law distribution such that the fraction of vertices *P*(*k*) having a degree *k* follows *P*(*k*) ∼ *k* ^−*γ*^ where *γ* ranges typically between 2 and 3 [41]. However, as noticed in [20], usually, notably in social networks, the actors do not have a global knowledge of the network. Consequently, the preferential attachment model is more likely to be local.

The second hypothesis underlying our model is that the membership to a community depends on the structural links and the attributes in such a way that firstly, there should be more edges between two vertices belonging to a same community than between vertices from different communities and secondly, two vertices belonging to the same community are likely to be more similar in terms of attributes as two vertices belonging to different communities.

Finally, our model is based on the homophily hypothesis, according to which two vertices are more likely to be connected if they share common characteristics and this property is verified inside the communities but also between communities [11, 42]. So, the more similar the vertices, the more likely connected they are.

### Model properties

Given these hypotheses, the proposed model allows us to generate attributed graphs having the following properties.

**P1. Local preferential attachment:** The local preferential attachment states that a vertex is more likely to create connections with vertices having a high degree and which are close [20].

**P2. Small world:** This property indicates that most vertices can be reached from every other through a small number of edges. According to [18], in a small world network, the average shortest path length is proportional to the logarithm of the number of vertices. The diameter can also be used to evaluate the small word property since it is defined as the maximum distance between any two vertices, where the distance is the minimum number of edges on the path from one vertex to the other one. It has been shown that real networks exhibit very small diameters, notably the well-known “six-degrees of separation” [43, 44].

**P3. Community structure:** A community structure appears when vertices can be grouped in a way such that vertices in a group are more connected to vertices in the same group compared to other vertices. While there is no formal definition of a network community, several measures have been proposed to control the community structure. In this article, we consider the modularity measure from [45]. Moreover, the average clustering coefficient from [18] is given as an indication of the transivity in the network.

**P4. Community homogeneity:** This property occurs when the vertices inside a community are more similar according to their attribute values compared to vertices in a different community. To measure this property, one can use the within inertia ratio. Given 𝓟, a partition of vertices and *d*(*v*_1_,*v*_2_), the euclidean distance between the real attribute vectors associated with the vertices *v*_1_ and *v*_2_, the within inertia ratio is $\frac{\sum_{C\in 𝓟}(P_{C}\sum_{v\in C}d{\left(v,g_{C}\right)}^{2})}{\sum_{v\in 𝓥}d{\left(v,g\right)}^{2}}$ where *g*_*C*_ is the center of gravity of the vertices in *C*, *P*_*C*_ the weight of *C* and *g* is the center of gravity of all the vertices.

**P5. Homophily:** This property is verified in networks where similar vertices according to their properties tend to be more connected than dissimilar vertices. To measure this property, we adapted the test introduced by [46] for numeric attributes. This test compares an *expected homophily measure* corresponding to the probability for two vertices to be similar with an *observed homophily measure* defined as the probability that two linked vertices are similar. If the expected measure is significantly less than the observed measure, then there is evidence for homophily.

### Model parameters

The previously described network properties can be controlled by the user using the model parameters summarized in Table 1.

**Table 1:** Description of the generator parameters.

**Parameter**	**Description**
*N* ∈ ℕ^+^	Number of vertices
$E_{wth}^{max}\in {\mathbb{N}}^{+}$	Maximum within (community) edges added to a new vertex
$E_{btw}^{max}\in \{0,\ldots ,E_{wth}^{max}\}$	Maximum between (community) edges added to a new vertex
*MTE* ∈ ℕ	Minimum number of edges in the resulting graph
𝓐 = {*σ*_1_,…,*σ*_∣𝓐∣_}	A set of attribute descriptors, i.e., standard deviation.
*K* ∈ ℕ^+^	Number of communities
*θ* ∈ [0, 1]	Threshold for community attributes homogeneity
*NbRep* ∈ ℕ^+^	Maximum number of community representatives

Description of the generator parameters

**Vertices:** Each generated graph has a fixed number of vertices controlled by the parameter *N* = ∣𝓥∣.

**Edges:** Three parameters control the edge insertion. Parameter $E_{wth}^{max}$ defines the maximum number of edges connecting a newly inserted vertex to other vertices in its community (i.e., within edges). To avoid disconnected vertices, $E_{wth}^{max}$ is required to be superior or equal to 1. On the other hand, parameter $E_{btw}^{max}$ defines the maximum number of edges connecting a newly inserted vertex to vertices out of its community (i.e., between edges). To ensure a community structure, $E_{btw}^{max}$ must range between 0 and $E_{wth}^{max}$. Note that when $E_{wth}^{max}$ equals to 0, the communities are completely disconnected. Finally, parameter *MTE* allows to set the minimal number of edges in the graph and consequently to control the density of the network. To reach this threshold, edges are inserted within the communities at the end of the process to reinforce the community structure by triadic closure *i.e.* by increasing the number of three fully connected vertices.

**Attributes:** The numeric attribute values associated with the vertices are generated according to normal laws defined by two parameters: ∣𝓐∣, the number of attributes and 𝓐, the set of attribute descriptors. An attribute descriptor is a standard deviation *σ*_*A*_, according which a component of the attribute vector associated with the vertices is generated following a normal law 𝓝(0,*σ*_*A*_). Note that using centered values does not change the distance between the vertices, so the mean is fixed to 0.

**Communities:** The number of communities is set using parameter *K*. Parameter *θ* allows to associate a vertex with a random community instead of a community formed by vertices sharing similar attributes. It models the hypothesis that community selection is not necessarily based on available attributes and thus seems stochastic. A low value of *θ* will generate highly homogeneous communities, while on the other hand, a higher value will decrease the community homogeneity. Finally, to reduce algorithm complexity, a set of representatives is built for each community to compare similarity with newcomers. Parameter *NbRep* defines the maximal number of representatives for each community.

### Algorithm

Our generator is named Algorithm 1 which stands for Attributed Networks with Communities Generator. It returns:

- An attributed graph 𝓖 = (𝓥,𝓔,𝓐), where 𝓥 is a set of vertices, 𝓔 a set of edges, 𝓐 a set of attributes and where *v*_*A*_ denotes the attribute vector associated with a vertex *v* ∈ *V*;
- A partition 𝓟 of 𝓥, corresponding to the generated attributed network, such that (1) ∀(*C*_1_,*C*_2_) ∈ 𝓟×𝓟,*C*_1_∩*C*_2_ = ∅; (2) ∀*C* ∈ 𝓟,*C* ≠ ∅; and (3) $\underset{C\in 𝓟}{\cup }C=𝓥$

#### Algorithm description

The main algorithm is divided into four parts. Vertices and attribute values are first generated, then communities are initialized. Vertices are then added by batch into the graph and finally an optional step allows to control the density of the network in ensuring a minimum number of edges (*MTE*). The vertex insertion step is also split into two subparts: community selection and edge insertion.

**1. Vertices and attribute generation:** An arbitrary set of *N* vertices is firstly generated (line 1, Algorithm Algorithm 1 in Table 2). For each of these vertices, ∣𝓐∣ real attribute values are generated according to normal distributions whose standard deviations are given by the attribute parameter 𝓐 (lines 2–3, Algorithm Algorithm 1 in Table 2).

**Table 2:** Algorithm 1.

**Input:** *N*, $E_{wth}^{max}$, $E_{btw}^{max}$, *MTE*, 𝓐, *K*, *θ*, *NbRep*
**Output:** An attributed graph 𝓖 = (𝓥,𝓔,𝓐)
**Output:** A partition 𝓟 = {*C*_1_,…,*C*_*K*_} of 𝓥
{Vertices and attributes generation}
1: 𝓥 ← an arbitrary set of *N* vertices
2: **for** *v* ∈ 𝓥 do
3: **for** *A* ∈ 𝓐 **do** *v*_*A*_ ← 𝓝(0,*σ*_*A*_)
{Community initialization}
4: Algorithm 2()
5: **for** *C* ∈ 𝓟 **do** *C*.*rep* ← *C*
6: *V*_*toAdd*_ ← 𝓥\∪_*C* ∈ 𝓟_ *C*
{Batch vertex insertion}
7: while *V*_*toAdd*_ ≠ ∅ do
8: **for** *v* ∈ *Sample*(*V*_*toAdd*_,*Rand*_*Uni*_({1,…,∣*V*_*toAdd*_∣})) do
9: **if** *Rand*_*Uni*_([0,1[) < *θ* **then** *C* ← *Rand*_*Uni*_(𝓟)
10: **else** $C\leftarrow \underset{C^{\prime }\in 𝓟}{\text{arg min}}dist(v,C^{\prime }.rep)$
11: Algorithm 3 (*v, C*)
12: C ← C ⋃ {v}
13: *V*_*toAdd*_ ← *V*_*toAdd*_\ {v}
14: **for** *C* ∈ 𝓟 **do** C.rep ← Sample(C, min(∣*C*∣, NbRep))
{Final edges insertion}
15: $MTE\leftarrow min(MTE,\sum_{C\in 𝓟}\frac{|C|\times (|C|-1)}{2})$
16: while ∣𝓔∣ < *MTE* do
17: *v* ← *Rand*_*Uni*_(*V*)
18: *E*_*tri*_ ← {{*v*_1_,*v*_2_} ∣ *v*_1_,*v*_2_ ∈ *neig*_*wth*_(*v*)∧*v*_1_ ≠ *v*_2_}\𝓔
19: 𝓔 ← 𝓔∪*Rand*_*Uni*_(*E*_*tri*_)

Algorithm 1

**2. Community initialization:** This initialization builds a first set of representatives of the communities, expected to have well separated attribute values (cf. property **P4**). It is described in Algorithm Algorithm 2 in Table 3 and the call to this algorithm is performed at line 4 in Algorithm Algorithm 1 in Table 2. First, a random sample *V*_*init*_ of *K* × *NbRep* vertices is drawn from 𝓥 to build the initial communities (line 1, Algorithm Algorithm 2 in Table 3). A KMedoids [47] clustering is then performed on these vertices to build *K* clusters but another clustering method could be used. The distance used in the KMedoids algorithm is euclidean. Each cluster provided by KMedoids corresponds to a community seed. As the generated clusters do not necessarily have the same cardinality, some vertices may be removed to ensure that each cluster has a cardinality equal to the smallest cluster generated (line 3, Algorithm Algorithm 2 in Table 3). Removed vertices are selected according to their distance to the cluster center of gravity to improve the homogeneity of the initial communities (cf. property **P4**) (line 5–6, Algorithm Algorithm 2 in Table 3). Edges are then inserted between vertices belonging to the same community to ensure a community structure (cf. property **P3**) (lines 8–12, Algorithm Algorithm 2 in Table 3). The number of within edges of a vertex is chosen uniformly using function *Rand*_*Uni*_ as well as its neighbors inside its community while respecting the constraint of maximum within edges given by $E_{wth}^{max}$. More precisely, given a set *S*, function *Rand*_*Uni*_(*S*) returns an element of *S* selected uniformly and randomly.

**Table 3:** Algorithm 2.

{Build initial communities}
1: *V*_*init*_ ← *Sample*(𝓥,*K* × *NbRep*)
2: 𝓟 ← *K Medoids*(*V*_*init*_,*K*)
3: $MinRep\leftarrow \underset{C\in 𝓟}{\text{min}}|C|$
4: **for** *C* ∈ 𝓟 **do**
{Resize the communities}
5: *g* ← Center of gravity of the elements in *C*
6: $C\leftarrow \underset{\underset{|C^{\prime }|=MinRep}{C^{\prime }\subseteq C}}{\text{arg min}}\underset{v\in C^{\prime }}{\sum }d(v,g)$
{Add initial within edges}
7: **for** *v* ∈ *C* **do**
8: $E_{wth}\leftarrow Rand_{Uni}(\{1,\ldots ,E_{wth}^{max}\})$
9: **repeat** *E*_*wth*_ **times**
10: *v*′ ← *Rand*_*Uni*_(*C*\{*v*})
11: 𝓔 ← 𝓔 ∪ {{*v*,*v*′}}
12: **end repeat**

Algorithm 2

**3. Batch vertex insertion:** This step iteratively adds vertices in the graph by batches of random size until all the vertices generated at step 1 have been inserted. The set of remaining vertices *V*_*toAdd*_ is first initialized (line 6, Algorithm Algorithm 1 in Table 2). Then, a batch of vertices is randomly sampled from *V*_*toAdd*_ (line 8, Algorithm Algorithm 1 in Table 2). For each vertex in a batch, the community selection and the edge insertion described in steps 3.a. and 3.b. are performed. Once all vertices in a batch have been inserted, community representatives are updated (line 14, Algorithm Algorithm 1 in Table 2). It is performed by randomly sampling *NbRep* vertices from each community *C* or ∣*C*∣ if the community contains less than *NbRep* vertices.

**3.a. Community selection:** The community of a vertex is chosen either randomly or as a function of its attributes and this choice depends on the parameter *θ*. When community membership is based on attributes that are not available, the community selection may seem stochastic and this is modeled by the random choice (line 9, Algorithm Algorithm 1 in Table 2). When the community selection is based on observed attributes, it respects the homophily property **P5** in such a way that the assignment to a community considers not only the average distance between the vertex and all the representatives of this community, but also its distance with the most similar representative (line 10, Algorithm Algorithm 1 in Table 2). The underlying assumption is that a single vertex can be more attractive than a set of vertices. Thus, the vertex *v* is affected to the community *C* such that *dist*(*v*,*C*) is minimized where *dist*(*v*,*C*) is defined by $\sum_{v^{\prime }\in C}\frac{d(v,v^{\prime })}{|C|}+\underset{v^{\prime }\in C}{\text{min}}d(v,v^{\prime })$. Finally, the vertex is added to the selected community *C* (line 12, Algorithm Algorithm 1 in Table 2).

**3.b. Edge insertion:** The vertex is then connected to the other vertices with function Algorithm 3 (line 11, Algorithm Algorithm 1 in Table 2). Its within degree (*E*_*wth*_) and its between degree (*E*_*bth*_) are firstly selected with the function ***Rand*_*PL*_** according to a power law with respect to the parameters $E_{wth}^{max}$ and $E_{btw}^{max}$ given by the user (lines 1 and 5, Algorithm Algorithm 3 in Table 4). The function *Rand*_*PL*_(*m*) returns a natural number belonging to {1,…,*m*} randomly selected using the probability density function $f:x\mapsto \frac{x^{-2}}{\sum_{i=1}^{m}i^{-2}}$. Then, the neighbors inside the community are chosen with the function *Rand*_*EdgeWth*_ in order to ensure the local preferential attachment **P1**. The function *Rand*_*EdgeWth*_(*V*) returns a vertex *u* from *V* randomly selected according to the probability density function $f:u\mapsto \frac{deg(u)}{\sum_{u^{\prime }\in V}deg(u^{\prime })}$ where *deg*(*u*) denotes the degree of vertex *u*. The other neighbors are selected with function *Rand*_*EdgeBtw*_ among the representatives of the other communities in such a way that they are more likely to be connected if they are similar to the vertex (cf. property **P5**). Indeed, the function *Rand*_*EdgeBtw*_(*v*,*V*) returns a vertex *u* from *V* according to the probability density function $f:u\mapsto \frac{d{\left(v,u\right)}^{-1}}{\sum_{u^{\prime }\in V}d{\left(v,u^{\prime }\right)}^{-1}}$.

**Table 4:** Algorithm 3.

**Input:** *v*, *C*
{Within edges}
1: $E_{wth}\leftarrow Rand_{PL}(min(|C|,E_{wth}^{max}))$
2: **while** *deg*_*wth*_(*v*) < *E*_*wth*_ **do**
3: *v* ^′^ ← *Rand*_*EdgeWth*_(*C*\*neig*_*wth*_(*v*))
4: 𝓔 ← 𝓔∪{{*v*,*v* ^′^}}
{Between edges}
5: $E_{btw}\leftarrow Rand_{PL}(min(E_{btw}^{max},E_{wth})+1)-1$
6: **while** *deg*_*btw*_(*v*) < *E*_*btw*_ **do**
7: $v^{\prime }\leftarrow Rand_{EdgeBtw}(v,\underset{\underset{C^{\prime }\neq C}{C^{\prime }\in 𝓟}}{\cup }C^{\prime }.rep)$
8: 𝓔 ← 𝓔∪{{*v*,*v* ^′^}}

Algorithm 3

**4. Final edges insertion:** The last step occurs only if the number of edges added at steps 2 and 3.b. does not reach *MTE*, the minimum number of edges in the resulting graph, fixed by the user. As the edges are only inserted within communities, this step allows reinforcing the community structure and increasing the average clustering coefficient (cf. **P3**). The insertion is performed as follows. A vertex *v* is selected at random (line 17, Algorithm Algorithm 1 in Table 2) and an edge is inserted between two distinct neighbors of *v* in its community which are not already connected (line 18–19, Algorithm Algorithm 1 in Table 2, where the set of neighbors of *v* in its community is denoted *neig*_*wth*_(*v*)). Several triangle closing models have been proposed in [20]. In our model, we retained the random one as it has been demonstrated in [20] that despite its simplicity, it gives good results.

This process is repeated until the graph contains at least *MTE* edges or until each community forms a clique (line 16, Algorithm Algorithm 1 in Table 2). Indeed, the real maximum number of edges corresponds to the case where each community is a clique. In that case given a partition *P*, *MTE* is $\sum_{C\in 𝓟}\frac{|C|\times (|C|-1)}{2}$.

## Experimental results

### Study of parameters impact

**Fig 1:**
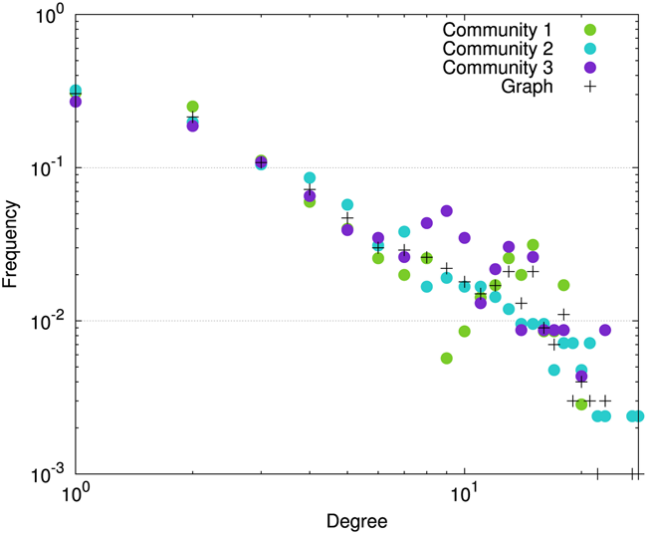
Vertices degree distribution in a graph generated using the reference parameter setting (log-log scale).

The aim of the first set of experiments is to demonstrate how, starting from a reference graph, the parameters can be used to either weaken or strengthen the community structure. The reference graph is generated using the following parameters: *K* = 3, *N* = 1,000, 𝓐 = {1,7}, *θ* = 0, *NbRep* = 100, $E_{wth}^{max}=6$, $E_{btw}^{max}=3$, and *MTE* = 0. The degree distributions of the vertices in the network (black dots) as well as within communities (colored dots) for the reference graph are given in Fig 1.

In the following set of experiments unless stated otherwise the parameters used to generate a graph correspond to those of the reference graph. The values of the measure plotted for different values of each parameter are the mean and standard deviation of the measure computed on 10 generated graphs with the same setting.

#### Community structure degradation

**Fig 2:**
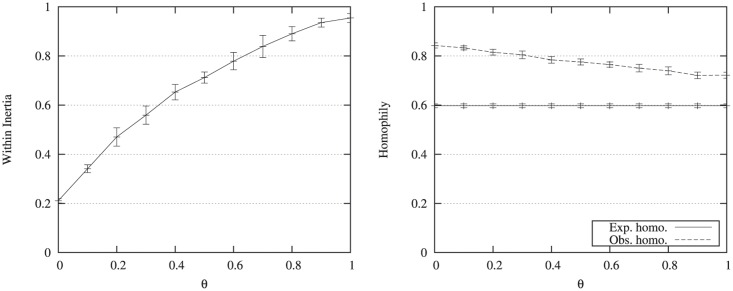
Community homogeneity measures for *θ* varying. The evolution of the within inertia is presented on the left side. The evolution of the homophily is presented on the right side.

**Fig 3:**
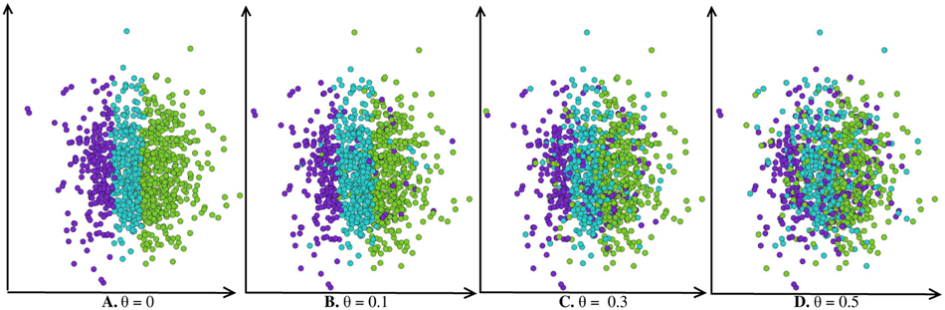
Distribution of the vertices for different values of *θ*. Each point corresponds to the attribute values in ℝ^2^ of a vertex along two axis. Colors correspond to communities. **A.** corresponds to *θ* = 0. **B.** corresponds to *θ* = 0.1. **C.** corresponds to *θ* = 0.3. **D.** corresponds to *θ* = 0.5.

As mentioned in the introduction, the community structure in an attributed graph relates to both the edges density and the attribute homogeneity inside the communities. To degrade the community homogeneity, we ran experiments in varying the values of the parameter *θ* which controls the probability to randomly assign a vertex to a community. Table 5 and Fig 2 present homogeneity measures for different values of *θ*. As expected, the increasing within inertia and decreasing observed homophily (for a constant expected homophily equal to 0.6) indicate that the homogeneity property is weakened. This behavior is also in agreement with the results presented in Fig 3 which plots the distribution in ℝ^2^ of the attribute values along two axis (one for each attribute). The higher the value of *θ*, the more heterogeneous the communities are.

**Table 5:** Community homogeneity measures for *θ* varying.

	*θ* = 0	*θ* = 0.1	*θ* = 0.3	*θ* = 0.5	*θ* = 1
Observed homophily	0.86	0.84	0.81	0.78	0.73
Expected homophily	0.60	0.60	0.60	0.60	0.60
Within inertia	0.22	0.37	0.59	0.72	0.96

Community homogeneity measures for *θ* varying

**Fig 4:**
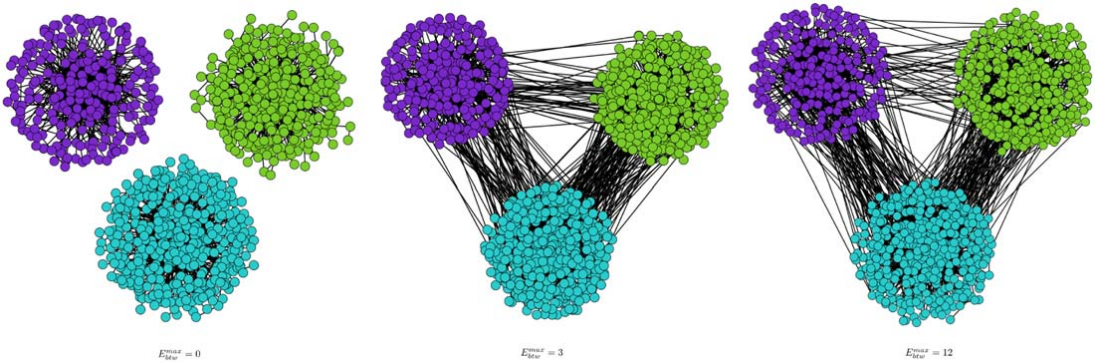
Example of graphs with generated varying $E_{btw}^{max}$.

**Fig 5:**
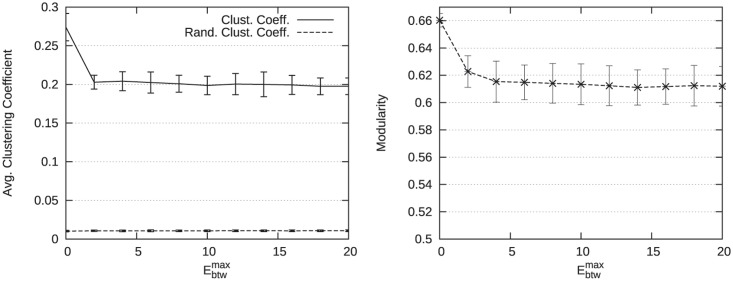
Evolution of structural measures for $E_{btw}^{max}$ varying. $E_{wth}^{max}$ is fixed to 20 to ensure that the number of within edges remains higher than the number of between edges. The evolution of the average clustering coefficient is presented on the left side. The evolution of the modularity is presented on the right side.

In the aim to degrade the community structure in such a way to have a number of within edges lower than the number of between edges, we performed experiments in varying the values of the parameter $E_{btw}^{max}$ which controls the maximum number of edges connecting a new vertex to vertices out of its community. Table 6 presents several structural measures for $E_{btw}^{max}\in \{0,3,12\}$ and $E_{wth}^{max}=14$. As shown in Fig 4 presenting the corresponding graphs, the number of edges between the communities increases. Fig 5 presents the average clustering coefficient (left side) and the modularity (right side) for $E_{btw}^{max}$ ranging between 0 and 20 and $E_{wth}^{max}=20$ to respect $E_{btw}^{max}\leq E_{wth}^{max}$. As expected, these results indicate that the community structure is degraded when parameter $E_{btw}^{max}$ increases. One can also notice that even if the community structure is degraded, the obtained average clustering coefficient remains higher than the one obtained in an Erds-Renyi random graph having the same number of vertices and edges (i.e., the random clustering coefficient measure).

**Table 6:** Structural measures for $E_{btw}^{max}$ varying and $E_{wth}^{max}=14$.

	$E_{btw}^{max}=0$	$E_{btw}^{max}=3$	$E_{btw}^{max}=12$
Modularity	0.66	0.59	0.57
Average clustering coefficient	0.12	0.09	0.08
Expected clustering coefficient	0.01	0.01	0.01
Average degree	6.02	6.45	6.56
Average shortest path	-	4.06	3.98
Diameter	-	8	9
Within Edges	3,010	2,986	3,002
Between Edges	0	238	279
Number of edges	3,010	3,224	3,281

Structural measures for $E_{btw}^{max}$ varying and $E_{wth}^{max}=14$

Moreover, in experiments where the attributes and the relations are degraded, using a tuning for the parameters *θ* = 0.5, $E_{btw}^{max}=12$ and $E_{wth}^{max}=14$, the measures confirm the degradation both on the community homogeneity and community structure. In particular, regarding the attribute homogeneity, the within inertia is 0.74 and the observed homophily is 0.81. For structural measures, the average clustering coefficient decreases to 0.08 and the modularity to 0.58.

#### Improving community structure

Opposite to the previous set of experiments, the community structure of the generated graphs can be improved compared to the reference graph. However, as the community homogeneity is already high in the reference graph (i.e., *θ* = 0), we cannot improve it and we consider the community structure based on links. We increased the number of within edges while keeping a fixed number of between edges. Two parameters allow to control the number of within edges, $E_{wth}^{max}$ and *MTE* which correspond respectively to the maximum number of edges connecting an inserted vertex to vertices in its community and the minimum number of edges in the final graph.

**Fig 6:**
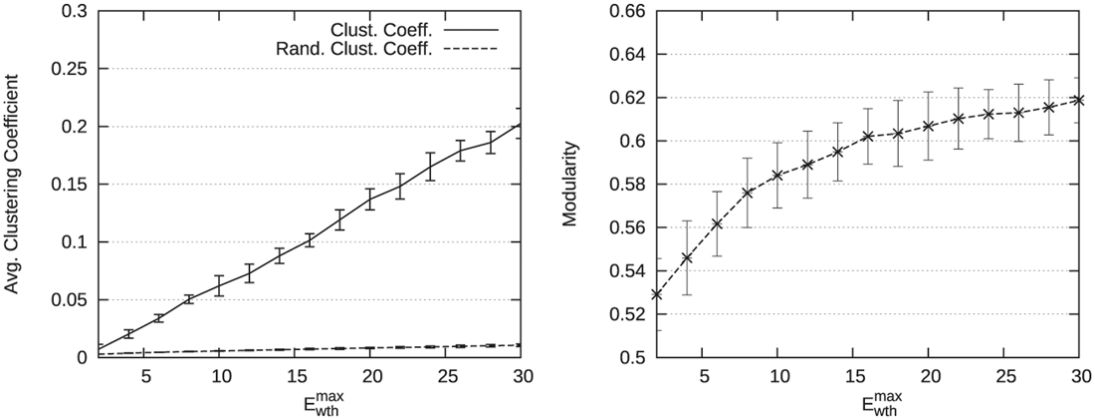
Evolution of structural measures for $E_{wth}^{max}$ varying. The evolution of the average clustering coefficient is presented on the left side. The evolution of the modularity is presented on the right side.

Structural measures for increasing values of parameter $E_{wth}^{max}$ are presented in Table 7 for $E_{wth}^{max}\in \{6,10,14,20\}$ and Fig 6 for $E_{wth}^{max}$ ranging between 2 and 30. The average clustering coefficient and the modularity increase when $E_{wth}^{max}$ increases, which seems to indicate a stronger community structure. Unsurprisingly, the average shortest path length and the diameter are also decreasing while the average degree is increasing when the number of edges grows.

**Table 7:** Structural measures for $E_{wth}^{max}$ varying.

	$E_{wth}^{max}=6$	$E_{wth}^{max}=10$	$E_{wth}^{max}=14$	$E_{wth}^{max}=20$
Modularity	0.56	0.58	0.59	0.60
Average clustering coefficient	0.03	0.06	0.09	0.15
Expected clustering coefficient	0.00	0.01	0.01	0.01
Average degree	4.49	5.67	6.45	8.18
Average shortest path	4.62	4.27	4.06	3.90
Diameter	11	9	8	8
Within Edges	2,037	2,610	2,986	3,862
Between Edges	210	223	238	230
Number of edges	2,247	2,833	3,224	4,092

Structural measures for $E_{wth}^{max}$ varying

**Fig 7:**
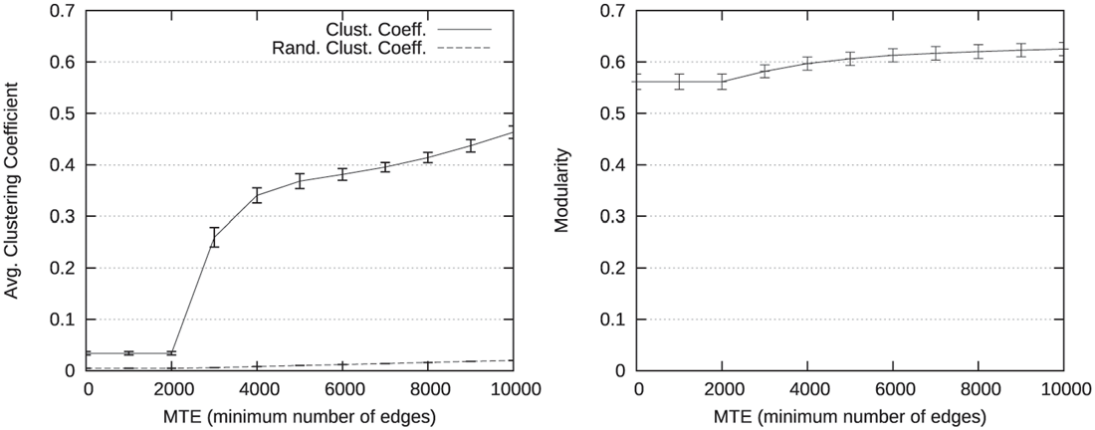
Evolution of structural measures for *MTE* varying. The evolution of the average clustering coefficient is presented on the left side. The evolution of the modularity is presented on the right side.

Results presented in Fig 7 for increasing values of the parameter *MTE* show a similar behavior regarding the modularity. However, the clustering coefficient reaches higher values for similar numbers of edges. In particular, when $E_{wth}^{max}=20$, the number of edges is approximately 4,000 and the average clustering coefficient is 0.15 in Table 7, while for *MTE* = 4000 it is 0.33 in Fig 7. Consequently, it seems that parameter *MTE* improves more significantly the community structure compared to parameter $E_{wth}^{max}$.

### Scalability

This second set of experiments aims firstly at studying the impact of increasing the number of vertices on the graph structure and secondly at studying runtime evolution.

**Fig 8:**
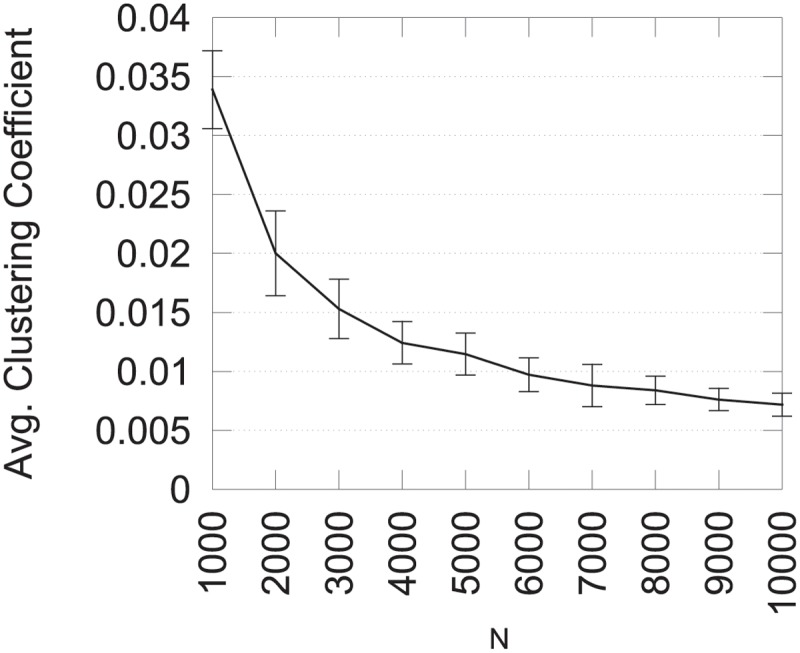
Average clustering coefficient for varying *N*.

**Fig 9:**
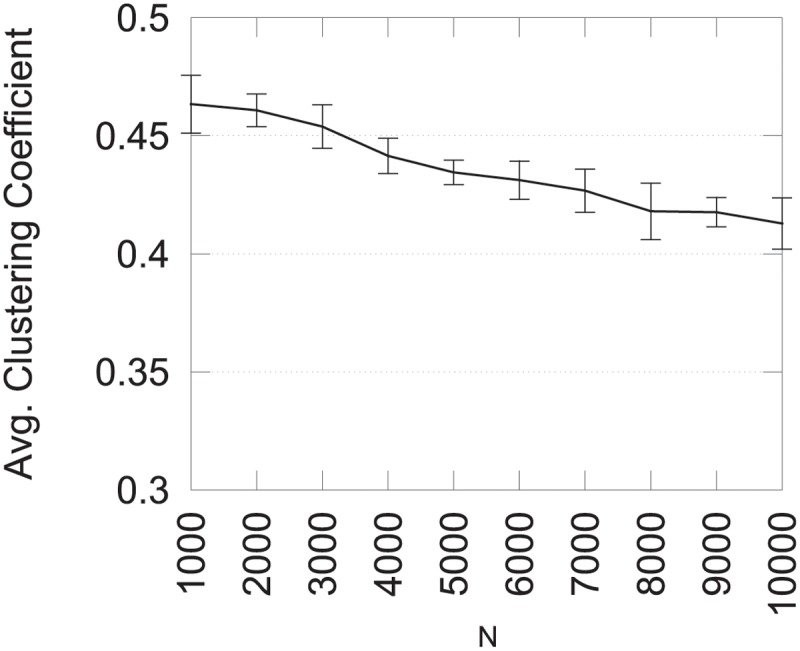
Average clustering coefficient for varying *N* and *MTE* = 10×*N*.

To study the first aspect, we computed the average clustering coefficient on 10 graphs for different values of the parameter *N*. Results are presented in Fig 8 and show that the average clustering coefficient decreases when the graph size increases. This behavior is due to the edge insertion process since the probability to add an edge which closes a triangle is lower for large number of vertices. To maintain a high clustering coefficient, it is possible to increase parameter *MTE* along with the number of vertices. Results where *MTE* = *N*×10 for varying number of vertices are presented in Fig 9. Using these parameters, the average clustering coefficient remains relatively stable.

**Fig 10:**
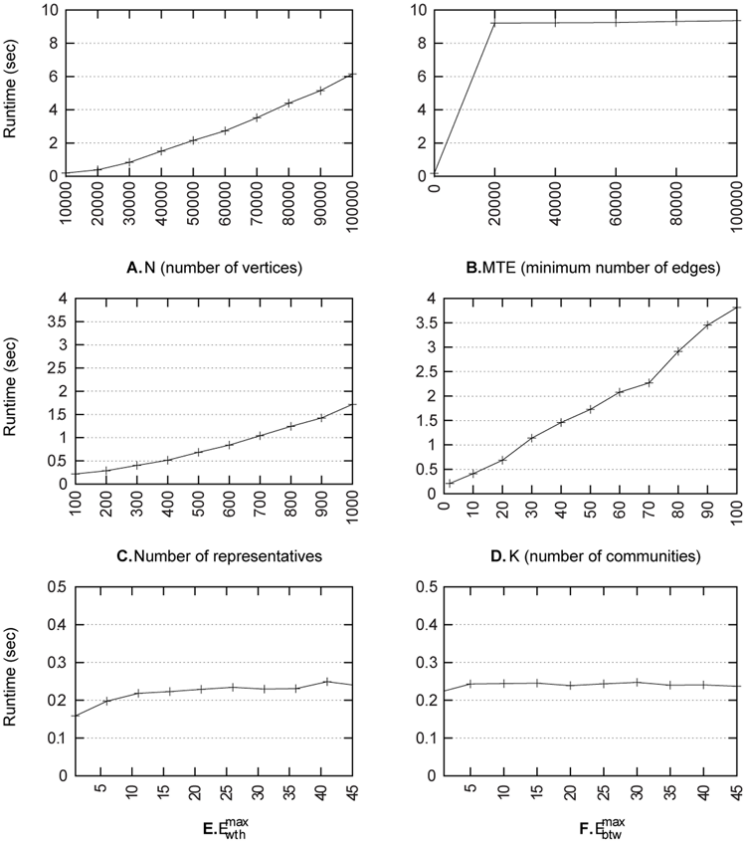
Generation runtimes for varying parameters. **A.** corresponds to varying number of vertices. **B.** corresponds to varying *MTE*. **C.** corresponds to varying number of representatives. **D.** corresponds to varying number of communities. **E.** corresponds to varying $E_{wth}^{max}$. **E.** corresponds to varying $E_{btw}^{max}$.

Runtime measures were computed using a standard computer running GNU/Linux with 8 Gb of main memory and an Intel® Core i5 3.2GHz CPU. Fig 10 presents generation runtime for different parameter settings starting with default parameters set as *K* = 3, *N* = 10,000, 𝓐 = {1,7}, *θ* = 0, *NbRep* = 100, $E_{wth}^{max}=6$, $E_{btw}^{max}=3$, and *MTE* = 0. For each setting, only one parameter is varied. The algorithm seems to scale linearly w.r.t. the parameters *N*, *NbRep*, and *K*. The parameters $E_{wth}^{max}$ and $E_{btw}^{max}$ seems to have very little impact on runtime. Finally, the runtime measure evolution in function of the parameter *MTE* increases by step and Fig 10B. presents one step for the first values of *MTE*. That is because most of the computation consists in finding the edges which can be added and it does not depend on the value of parameter *MTE*. These results indicate that the proposed approach is able to generate large graphs. In particular, generating a graph having one million vertices requires 467 seconds.

## Output, interface and evaluation criterion

**Fig 11:**
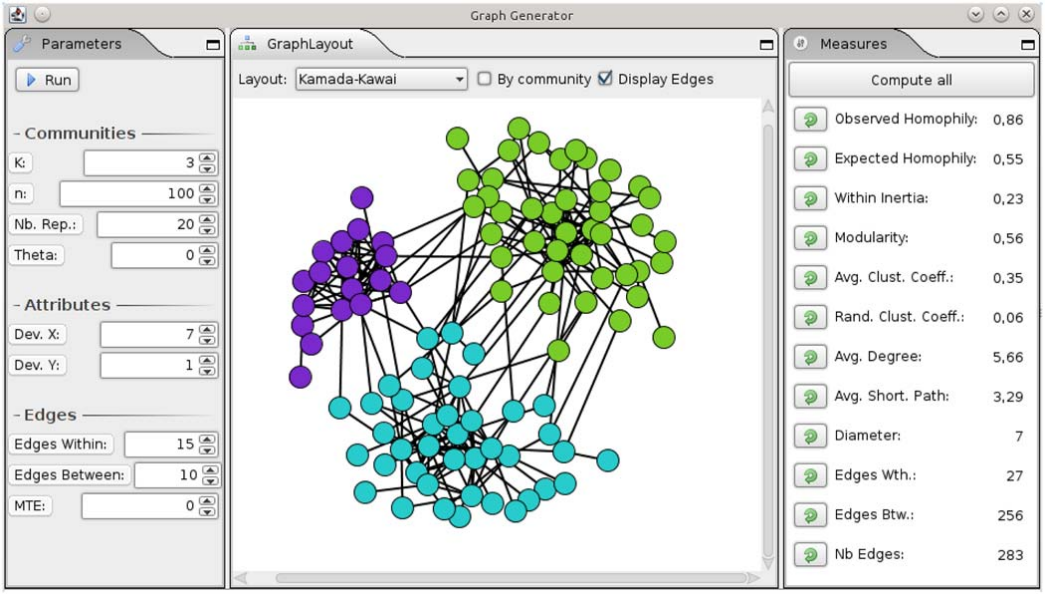
User interface of the generator.

Algorithm 1 is available at the url http://perso.univ-st-etienne.fr/largeron/ANC_Generator/. It is a free software distributed under the terms of the GNU General Public Licence (version 3). It is implemented in Java and it can be executed on any platform with a Java virtual machine. A screen copy of the user interface of the generator is presented in Fig 11. It is formed by three views. On the left side, the user selects the generator parameters presented in Table 1. The central part displays the generated graph using either a layout based on the graph structure (e.g., Kamada-Kawaï) or based on the attribute values for ∣𝓐∣ = 2. The right side of the interface presents the measures corresponding to the generated graph.

- The difference between the expected and observed homophily allows to measure if similar vertices according to the attributes tend to be more connected than dissimilar vertices (cf **P5**);
- The within inertia measures the dispersion of the attribute values inside the communities (cf. **P4**). A low within inertia indicates that the communities are highly homogeneous with regard to the attribute values;
- The modularity defined by Newman [45] (cf. **P3**);
- The network average clustering coefficient is a measure of the clustering tendency of the network (cf. **P3**). This observed value can be compared with an expected value computed on a random graph having the same vertex set: an observed value higher than the expected value confirms the community structure;
- The average degree is the average number of neighbors of a vertex (cf. **P1**);
- The average shortest path is the average minimum number of hops required to reach two arbitrary vertices (cf. **P2**). It is not computed when the graph is formed by several disconnected components (i.e., $E_{btw}^{max}=0$);
- The diameter is the longest shortest path between any two vertices (cf. **P2**);
- The number of between edges and the number of within edges are respectively the number of edges connecting vertices in the same community and the number of edges connecting vertices in different communities (cf. **P3**);
- The number of edges in the graph, i.e., ∣𝓔∣.

## Conclusion

In this article we proposed a network generator having a known community structure and where vertices are associated with numeric attributes. This generator constructs graphs with real networks properties both with respect to the structure, i.e., local preferential attachment, small world property, community structure, and with respect to the attributes, i.e., homogeneous communities and homophily. Several parameters allow the user to control the structure of the graph. We performed experiments to study how these parameters can impact the generated graph and demonstrated that they allow a degradation of the structure of the network on the one hand, and the deterioration of the homogeneity of the communities on the other hand. Regarding runtime, our generator is able to provide graphs having one million vertices. A possible extension of this work is to generate graphs where the attributes can be either numeric or categoric.

## References

[1] Newman ME. Networks: An Introduction. Oxford University Press, Inc.; 2010

[2] Fortunato S, Castellano C. Community structure in graphs In: Computational Complexity, Springer; 2012 p. 490–512.

[3] Orman GK, Labatut V. The effect of network realism on community detection algorithms. In: International Conference on Advances in Social Networks Analysis and Mining. 2010. p. 301–305.

[4] Lancichinetti A, Fortunato S, Radicchi F. Benchmark graphs for testing community detection algorithms. Physical Review E. 2008;78: 046110 10.1103/PhysRevE.78.046110 18999496

[5] Lancichinetti A. Evaluating the performance of clustering algorithms in networks In: Dynamics On and Of Complex Networks, Springer, volume 2 2013 p. 143–158.

[6] Gustafsson M, Hörnquist M, Lombardi A. Comparison and validation of community structures in complex networks. Physica A: Statistical Mechanics and its Applications. 2006; 367: 559–576. 10.1016/j.physa.2005.12.017

[7] Lancichinetti A, Fortunato S. Community detection algorithms: A comparative analysis. Physical Review E. 2009; 80: 056117 10.1103/PhysRevE.80.056117 20365053

[8] Orman GK, Labatut V, Cherifi H. Qualitative comparison of community detection algorithms. In: International Conference on Digital Information and Communication Technology and Its Applications. volume 167 2011 p. 265–279. 10.1007/978-3-642-22027-2_23

[9] Moussiades L, Vakali A. Benchmark graphs for the evaluation of clustering algorithms. In: International Conference on Research Challenges in Information Science (RCIS). 2009. p. 197–206.

[10] Zhou Y, Cheng H, Yu JX. Graph clustering based on structural/attribute similarities. Proceedings of the VLDB Endowment (pVLDB) 2009; 2: 718–729. 10.14778/1687627.1687709

[11] McPherson M, Smith-Lovin L, Cook JM. Birds of a feather: Homophily in social networks. Annual Review of Sociology. 2001; 27: 415–444. 10.1146/annurev.soc.27.1.415

[12] Goldenberg A, Zheng AX, Fienberg SE, Airoldi EM. A survey of statistical network models. Found Trends Mach Learn. 2010; 2: 129–233. 10.1561/2200000005

[13] Vázquez A. Growing network with local rules: Preferential attachment, clustering hierarchy, and degree correlations. Physical Review E. 2003; 67: 056104 10.1103/PhysRevE.67.056104 12786217

[14] Chakrabarti D, Faloutsos C. Graph mining: Laws, generators, and algorithms. ACM Computing Surveys. 2006; 38: 1–78. 10.1145/1132952.1132954

[15] Newman ME, Watts DJ, Strogatz SH. Random graph models of social networks. Proceedings of the National Academy of Sciences of the United States of America. 2002; 99: 2566–2572. 10.1073/pnas.012582999 11875211 PMC128577

[16] Erdős P, Rényi A. On the evolution of random graphs. Publication of the mathematical institute of the Hungarian academy of sciences. 1960; 5: 17–61.

[17] Newman MEJ, Girvan M. Finding and evaluating community structure in networks. Physical Review E. 2004; 69: 026113 10.1103/PhysRevE.69.066133 14995526

[18] Watts DJ, Strogatz SH. Collective dynamics of ‘small-world’ networks. Nature. 1998; 393: 440–442. 10.1038/30918 9623998

[19] Barabási AL, Albert R. Emergence of scaling in random networks. Science. 1999; 286: 509–512. 10.1126/science.286.5439.509 10521342

[20] Leskovec J, Backstrom L, Kumar R, Tomkins A. Microscopic evolution of social networks. In: ACM SIGKDD International Conference on Knowledge Discovery and Data Mining (KDD). 2008. p. 462–470.

[21] Leskovec J, Kleinberg J, Faloutsos C. Graphs over time: densification laws, shrinking diameters and possible explanations. In: ACM SIGKDD International Conference on Knowledge Discovery in Data Mining (ICDM). 2005. p. 177–187.

[22] Leskovec J, Kleinberg J, Faloutsos C. Graph evolution: Densification and shrinking diameters. ACM Transactions on Knowledge Discovery from Data (TKDD). 2007; 1: 2 10.1145/1217299.1217301

[23] Leskovec J, Chakrabarti D, Kleinberg J, Faloutsos C, Ghahramani Z. Kronecker graphs: An approach to modeling networks. The Journal of Machine Learning Research. 2010; 11: 985–1042.

[24] Benson AR, Riquelme C, Schmit S. Learning multifractal structure in large networks. In: Proceedings of the 20th ACM SIGKDD International Conference on Knowledge Discovery and Data Mining (KDD). 2014.

[25] Palla G, Lovsz L, Vicsek T. Multifractal network generator. Proceedings of the National Academy of Sciences. 2010; 107: 7640–7645. 10.1073/pnas.0912983107 PMC2867894 20385847

[26] Girvan M, Newman ME. Community structure in social and biological networks. Proceedings of the National Academy of Sciences. 2002; 99: 7821–7826. 10.1073/pnas.122653799 PMC122977 12060727

[27] Lancichinetti A, Fortunato S. Benchmarks for testing community detection algorithms on directed and weighted graphs with overlapping communities. Physical Review E. 2009; 80: 016118 10.1103/PhysRevE.80.016118 19658785

[28] Newman ME. The structure and function of complex networks. SIAM review. 2003; 45: 167–256. 10.1137/S003614450342480

[29] Orman GK, Labatut V. A comparison of community detection algorithms on artificial networks In: Discovery Science (DS). Springer 2009 p. 242–256.

[30] Orman GK, Labatut V, Cherifi H. Towards realistic artificial benchmark for community detection algorithms evaluation. International Journal of Web Based Communities. 2013; 9: 349–370. 10.1504/IJWBC.2013.054908

[31] Poncela J, Gómez-Gardeñes J, Floría LM, Sánchez A, Moreno Y. Complex cooperative networks from evolutionary preferential attachment. PLoS one. 2008; 3: e2449 10.1371/journal.pone.0002449 18560601 PMC2413409

[32] Kolda TG, Pinar A, Plantenga T, Seshadhri C. A scalable generative graph model with community structure. CoRR. 2013.

[33] Seshadhri C, Kolda TG, Pinar A. Community structure and scale-free collections of erdős-rényi graphs. Physical Review E. 2012; 85: 056109 10.1103/PhysRevE.85.056109 23004823

[34] Kim M, Leskovec J. Multiplicative attribute graph model of real-world networks. Internet Mathematics. 2012; 8: 113–160. 10.1080/15427951.2012.625257

[35] Wong LH, Pattison P, Robins G. A spatial model for social networks. Physica A: Statistical Mechanics and its Applications. 2006; 360: 99–120. 10.1016/j.physa.2005.04.029

[36] Gong NZ, Xu W, Huang L, Mittal P, Stefanov E, et al Evolution of social-attribute networks: Measurements, modeling, and implications using Google+ In: ACM Conference on Internet Measurement Conference (IMC). ACM 2012 p. 131–144.

[37] Akoglu L, Faloutsos C. Rtg: a recursive realistic graph generator using random typing. Data Mining and Knowledge Discovery (DMKD). 2009; 19: 194–209. 10.1007/s10618-009-0140-7

[38] Dang TA. Analysis of Communities in Social Networks. Ph.D. thesis, Université Paris 13. 2012

[39] Yang J, McAuley J, Leskovec J. Community Detection in Networks with Node Attributes. IEEE 13th International Conference on Data Mining. 2013. p. 1151–1156.

[40] Palla K, Knowles DA, Ghahramani Z. An infinite latent attribute model for network data. In: Proceedings of the 29th International Conference on Machine Learning (ICML). 2012. p. 1607–1614.

[41] Albert R, Barabási AL. Statistical mechanics of complex networks. Review of Modern Physics. 2002; 74: 47–97. 10.1103/RevModPhys.74.47

[42] Lazarsfeld PF, Merton RK. Friendship as a social process: A substantive and methodological analysis. Freedom and Control in Modern Society. 1954; 18: 18–66.

[43] Milgram S. The small-world problem. Psychology Today. 1967; 2: 60–67.

[44] Amaral LAN, Scala A, Barthélémy M, Stanley HE. Classes of small-world networks. Proceedings of the National Academy of Sciences. 2000; 97: 11149–11152. 10.1073/pnas.200327197 PMC17168 11005838

[45] Newman ME. Finding community structure in networks using the eigenvectors of matrices. Physical review E. 2006; 74: 036104 10.1103/PhysRevE.74.036104 17025705

[46] Easley D, Kleinberg J. Networks, Crowds and Markets: Reasoning about a Highly Connected World, Cambridge University Press, chapter Networks in their Surrounding Contexts. 2010. p. 85–118.

[47] Kaufman L, Rousseeuw P. Clustering by Means of Medoids Reports of the Faculty of Mathematics and Informatics. Faculty of Mathematics and Informatics 1987.

